# The Neutrophil Percentage–to–Albumin Ratio: An Emerging Biomarker Associated With Primary Stroke Risk in Hypertensive Patients

**DOI:** 10.1111/jch.70236

**Published:** 2026-03-31

**Authors:** Longguo Zhao, Vipin Kumar, Megumi Narisawa, Yanglong Li, Kai Meng, Guang Yang, Xian Wu Cheng

**Affiliations:** ^1^ Jilin Provincial Key Laboratory of Stress and Cardiovascular Disease Yanbian University Hospital Yanji Jilin PR China; ^2^ Department of Cardiology Nagoya University Graduate School of Medicine Nagoya Aichi Japan; ^3^ Department of Radiology Yanbian University Hospital Yanji Jilin PR China; ^4^ Department of Cardiology and Hypertension Yanbian University Hospital Yanji Jilin PR China

**Keywords:** blood pressure, neutrophil to albumin ratio, stroke, variability

**Abbreviations**
NPAR: The neutrophil percentage–to–albumin ratio

Stroke remains a leading cause of mortality and long‐term disability worldwide, and hypertension is among the most significant modifiable risk factors for both ischemic stroke and hemorrhagic stroke [1]. Despite the well‐documented advantages of blood pressure control and conventional vascular risk factor management, a significant percentage of primary strokes occur in individuals who are not considered high‐risk based solely on standard clinical predictors. This highlights the importance of refining primary prevention strategies through improved risk stratification and the identification of additional pathophysiological pathways contributing to cerebrovascular events. Inflammation is a critical driver of atherosclerotic plaque instability and thrombosis, thus prompting interest in inflammatory markers as potential predictors of cerebrovascular events. The neutrophil percentage‐to‐albumin ratio (NPAR) has emerged as a promising biomarker that integrates neutrophil‐related measures with serum albumin, offering a low‐cost and readily available indicator of systemic inflammation. Although the NPAR has demonstrated potential prognostic value in cardiovascular disease and diverse clinical settings (e.g., acute kidney injury and malignancy) and secondary‐stroke prevention [2, 3, 4]. Its role in predicting primary stroke among hypertensive patients remains unexplored.

In this issue of *The Journal of Clinical Hypertension*, Wang et al. examine the association between the NPAR, a readily accessible marker of systemic inflammation, and the risk of primary stroke among patients with hypertension [5]. They analyzed data from 13 848 individuals who had been enrolled in the China Stroke Primary Prevention Trial, a large‐scale, multi‐community randomized controlled trial evaluating folic acid supplementation for stroke prevention in hypertensive adults [6]. After the exclusion of subjects with missing baseline neutrophil percentage or albumin data, the final cohort was 5572 men and 8276 women (age 59.8 ± 7.6 years. The NPAR was calculated as (neutrophil percentage × 100)/albumin (g/dL). The subjects were stratified into quartiles based on this ratio. Over a median follow‐up period of 4.5 years, 371 (2.7%) subjects experienced a first stroke, defined as a nonfatal or fatal stroke (excluding subarachnoid hemorrhage and silent stroke).

One of the study's core findings is the association between higher NPAR values and an increased risk of stroke in individuals with hypertension. A Kaplan–Meier curve analysis also revealed that the cumulative incidence of stroke was consistently lower in the lowest NPAR quartile than in the upper three quartiles, further reinforcing the NPAR's prognostic value in this population. In Cox proportional hazards models that were adjusted for a comprehensive set of confounders (including age, sex, body mass index, blood pressure, homocysteine, cholesterol, and smoking status), the subjects in the NPAR quartiles Q2 (NPAR 10.7–12.0), Q3 (12.0–13.4), and Q4 (≥ 13.4) demonstrated a significantly higher risk of stroke compared to the reference group (Q1, < 10.7), with hazard ratios (HRs) at 1.76 (95%CI: 1.31–2.36, *p* < 0.001) (Figure 1), 1.54 (95%CI: 1.14–2.08, *p* = 0.005), and 1.57 (95%CI: 1.17–2.12, *p* = 0.003), respectively. Combining Q2, Q3, and Q4 into a single high‐NPAR group yielded a robust association (HR 1.62, 95%CI: 1.25–2.10, *p* <0.001), suggesting that NPAR values > 10.7 are linked to an increased stroke risk that is independent of traditional cardiovascular confounders.

**FIGURE 1:**
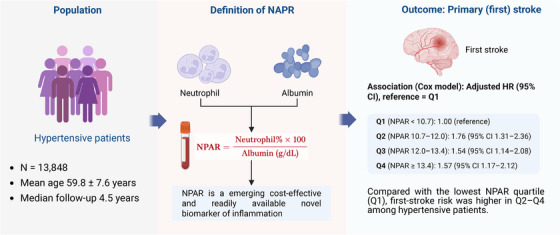
The neutrophil percentage‐to‐albumin ratio and first stroke risk in hypertensive patients.

Wang et al. also reported a significant sex‐specific difference in the association between the NPAR and stroke risk. Higher NPAR values strongly predicted stroke in women across both crude and adjusted models. In the adjusted analysis, the HRs were 2.36 (Q2), 2.01 (Q3), and 2.00 (Q4), and the combined HR was 2.13 (95%CI: 1.48–3.07, *p* < 0.001) for Q2–Q4 versus Q1. However, no significant association was observed in the men. This sex disparity (*p* for interaction = 0.035) suggests that distinct inflammatory mechanisms may contribute to the stroke pathogenesis in male and female hypertensive individuals. Wang et al. hypothesize that this difference may stem from the higher prevalence of traditional stroke risk factors (e.g., smoking and dyslipidemia) in men, which could mask the incremental predictive value of inflammatory biomarkers such as the NPAR. In contrast, the women in this cohort had fewer traditional risk factors, allowing the role of inflammation (as reflected by the NPAR) to emerge as a more prominent predictor.

These sex‐specific finding requires a closer examination, because they challenge the conventional approach to assessing cardiovascular risk. In the women in the Wang et al. study, elevated NPAR was associated with a more than doubled risk of stroke, while in the men, the association failed to reach statistical significance, despite the large number of males studied (*n* = 5572). However, it is important to consider alternative explanations for these findings that go beyond the masking effect of traditional risk factors. For example, sex hormones profoundly affect inflammatory pathways. The loss of estrogen's anti‐inflammatory properties after menopause could make women more susceptible to inflammation‐mediated vascular injury [7]. The mean age of the female subjects (59.2 years) suggests that most of them were postmenopausal, a period during which the cardiovascular risk accelerates dramatically in women. Additionally, the differences in inflammatory pathway activation, endothelial function, and vascular reactivity between the sexes could influence how systemic inflammation leads to cerebrovascular events [8]. The observational design of the Wang et al. study cannot definitively establish which mechanisms account for the sex difference, leaving this as a critical area for future mechanistic research.

Another notable observation reported by Wang et al. is the absence of a linear dose‐response relationship between NPAR quartiles and stroke risk. Although Q2, Q3, and Q4 were all associated with a significantly higher stroke risk compared to Q1, the HRs did not increase progressively with higher NPAR quartiles. NPAR values above the lowest quartile (Q1; < 10.7 in this cohort), may be associated with a level of systemic inflammation sufficient to contribute to pro‐atherogenic and pro‐thrombotic processes, potentially increasing stroke risk, though the precise biological mechanisms underlying this association remain to be established. Beyond this threshold, further increases in the NPAR did not demonstrate a progressively higher risk. Although this pattern is consistent with a threshold effect in which inflammation reaches a biologically impactful level, alternative explanations warrant consideration, including statistical variation, residual quartile‐specific confounding, and ceiling effects in risk assessment. Further mechanistic studies are needed to confirm whether this pattern reflects a true saturation of inflammatory pathways.

The biological basis of the NPAR as a predictor of stroke risk is supported by its composite nature, which incorporates two important indicators of systemic inflammation and metabolic health. Elevated neutrophil percentages are linked to atherosclerotic plaque instability and thromboembolic events [9]. In contrast, serum albumin is a negative acute‐phase reactant that decreases during inflammation [10]. Reduced albumin levels (hypoalbuminemia) reflect severe inflammation, increased capillary permeability, and an impaired nutritional status. These conditions are associated with an increased risk of cardiovascular complications [10]. Although the prognostic value of the NPAR has been validated in various clinical settings (including heart failure, acute myocardial infarction, atrial fibrillation, and peritoneal dialysis [2, 3, 4]), Wang et al.’s work is the first large‐scale study to demonstrate the NPAR's utility in primary stroke prevention in hypertensive individuals.

Despite its strengths, the study has several limitations that warrant consideration. First, the NPAR was calculated using a single baseline measurement, which may not capture long‐term fluctuations in inflammatory status. A longitudinal assessment of NPAR changes over time could improve our understanding of this ratio's dynamic relationship with stroke risk. Second, because the study is observational, it cannot establish causation. While the NPAR is associated with stroke risk, it remains unclear whether reducing the NPAR (e.g., through anti‐inflammatory treatments) would directly lower the stroke incidence. Third, residual or unmeasured confounding factors (e.g., dietary habits, physical activity, and medication use that were not captured in the baseline questionnaire) may have influenced the study's results. Finally, the cohort consisted of hypertensive Chinese aged 45–75 years, which limits the generalizability of the findings to other ethnic groups or age ranges. Future research should address these limitations by conducting larger prospective studies in diverse populations to validate the association between the NPAR and stroke risk. Interventional studies are also necessary to determine whether reducing the NPAR through lifestyle modifications (e.g., improved nutrition, smoking cessation) or pharmacological agents (e.g., statins, anti‐inflammatory drugs) would lower the stroke incidence, particularly among high‐risk females. Furthermore, although Wang et al. reported 10.7 as the threshold between the first and second NPAR quartiles, this value depends on the cohort and distribution and has not been validated as a clinically meaningful cutoff. Future studies should evaluate NPAR as a continuous predictor, derive candidate thresholds, and validate them externally to determine whether 10.7‐or another value‐provides reproducible discrimination and clinical utility for predicting stroke risk in hypertensive patients.

In conclusion, Wang et al.’s study provides robust evidence that a higher NPAR value is an independent predictor of a first stroke in hypertensive individuals, with a notably stronger association in women. The study emphasizes the importance of incorporating inflammatory biomarkers into stroke‐risk assessments and highlights the necessity of sex‐specific approaches to primary prevention. As an index that is easily obtainable from routine laboratory tests, the NPAR shows great potential for clinical application, providing a practical method to identify individuals at high risk and optimize preventive care. Although more research is needed to confirm causality and increase generalizability, the Wang et al. study is a significant step forward in addressing the residual burden of stroke in hypertensive populations.

## Author Contributions

Longguo Zhao wrote the first draft of the manuscript. Vipin Kumar and Megumi Narisawa drafted the Figure. Yanglong Li, Guang Yang, and Kai Meng edited the manuscript. Xian Wu. Cheng handled the funding and supervision.

## Conflicts of Interest

The authors declare no conflicts of interest.

## Data Availability

The authors have nothing to report.
